# Image generation technology for functional occlusal pits and fissures based on a conditional generative adversarial network

**DOI:** 10.1371/journal.pone.0291728

**Published:** 2023-09-19

**Authors:** Zhaodan Gu, Zhilei Wu, Ning Dai

**Affiliations:** 1 Jiangsu Automation Research Institute, Lianyungang, P.R. China; 2 College of Mechanical and Electrical Engineering, Nanjing University of Aeronautics and Astronautics, Nanjing, P.R. China; Shanghai Maritime University, CHINA

## Abstract

The occlusal surfaces of natural teeth have complex features of functional pits and fissures. These morphological features directly affect the occlusal state of the upper and lower teeth. An image generation technology for functional occlusal pits and fissures is proposed to address the lack of local detailed crown surface features in existing dental restoration methods. First, tooth depth image datasets were constructed using an orthogonal projection method. Second, the optimization and improvement of the model parameters were guided by introducing the jaw position spatial constraint, the *L*1 loss and the perceptual loss functions. Finally, two image quality evaluation metrics were applied to evaluate the quality of the generated images, and deform the dental crown by using the generated occlusal pits and fissures as constraints to compare with expert data. The results showed that the images generated using the network constructed in this study had high quality, and the detailed pit and fissure features on the crown were effectively restored, with a standard deviation of 0.1802mm compared to the expert-designed tooth crown models.

## 1. Introduction

Tooth defects refer to damaged or abnormal appearance and structure of dental hard tissues. They arise for various reasons, most frequently from dental restoration treatment. Tooth defects can destroy the normal occlusal relationship of teeth, leading to a decline in human digestive function, impaired phonation, and even facies, and they can induce diseases such as dental caries and periodontal disease [1, 2]. Therefore, the design of dental prostheses for tooth defects has long been a research focus in the field of stomatology.

Traditional prostheses are mainly designed and made by hand, and the process contains many steps, resulting in a long treatment cycle. Moreover, these methods are strongly influenced by the personal experience and technology used by dentists and cannot guarantee the accuracy of prostheses for defective teeth. The requirement of the patient to cooperate with multiple visits and adjustments throughout treatment also introduces additional inconvenience to the patient, and a full-crown prosthesis sample cannot be preserved after manufacturing, precluding bringing any experience to future diagnosis and treatment.

Today, widely applied dental restoration methods are mainly completed using computer-aided design/computer-aided manufacturing (CAD/CAM) design systems [3], which can eliminate some complicated steps in the design and manufacturing of traditional dental prostheses and shorten the treatment cycle. Despite the numerous advantages of CAD/CAM technology over traditional manual restoration, dental CAD systems mainly use standard teeth in their corpus databases and make adjustments through a series of deformation algorithms to design the final prostheses [4, 5]. In addition, due to the many artificial interactive operations required during the design, the prosthesis will lack local detailed features and differ significantly from natural teeth in morphology, and the prosthesis quality cannot be guaranteed.

With the development of artificial intelligence, it has been extensively applied and shown to be valuable in many fields, and many researchers have attempted to apply this technology to the field of dental restoration, for example, using deep learning methods for dental diagnosis, teeth segmentation, and dental restoration. Son et al. [6] proposed a novel framework called Dental Diagnosis System (DDS) to assist dental clinicians in their professional work. To address great differences in the gingival margin morphology between different individuals and different dental positions, Tian et al. [7] present a deep adversarial network-driven gingival margin line reconstruction (GMLR) framework to automatically obtain the personalized gingival contour for a partially edentulous patient. Wu et al. [8] proposed a model-based method to segment teeth and to measure parameters for orthodontic dentists in treatment planning and outcome evaluation. The parameters can be easily calculated by the presented model landmarks. Zhao et al. [9] designed a two-stream graph convolutional network, called TSGCN, to segment individual teeth from the intra-oral scanner images. The results demonstrate the superiority of proposed method, especially for the practically challenging cases. In order to detect early-stage dental caries on patient’s teeth OCT images, Karimian et al. [10] utilized a deep convolutional neural network(CNN) to classify oral tissues, achieving detection accuracy ranging from 97.93 to 99.85%. Liu et al. [11] developed an automatic diagnosis model trained by MASK R-CNN for the detection and classification of 7 different dental diseases with the diagnosis accuracy of them reaching up to 90%. Hatvani et al. [12] adopted a subpixel CNN and a U-net to improve the resolution of 2-D CBCT image slices of ex vivo teeth. Zhang et al. [13] have proposed a joint CMF bone segmentation and landmark digitization (JSD) framework via a context-guided multi-task FCN. Lang et al. [14] proposed a deep learning method to gradually and jointly localize a total of 105 CMF land-marks from CBCT images. The results show that the proposed method outper-forms state-of-the-art methods quantitatively and qualitatively. Lian et al. [15] proposed an end-to-end deep learning network for automated labeling of tooth model surfaces, named MeshSegNet. Additionally, Zhang et al. [16] proposed a convolutional neural network based on sparse octrees, which achieved automatic segmentation and extraction of the margin line of dental preparations. Wu et al. [17] proposes a two-stage framework based on mesh deep learning (called TS-MDL) for joint tooth labeling and land-mark identification on raw intraoral scans. The results suggest that it has the potential to be utilized in orthodontic applications. Xiao et al. [18] developed a geometric deep learning framework for reference CMF bony shape estimation in orthognathic surgical planning that reduces experience-dependent variability and improves planning accuracy and efficiency. Ma et al. [19] introduced FSC-Net for the rapid simulation of the postoperative facial appearance for efficient orthognathic surgical planning. Evaluation results demonstrated that FSC-Net significantly accelerates simulation with performance comparable to a SOTA FEM method. However, these methods are designed for specific dental tasks and are not suitable for dental crown occlusal surface reconstruction. Meanwhile, the successful applications of the aforementioned methods indicate that deep learning has tremendous potential in the field of dental crown restoration. Recently, some previous studies have employed generative models for reconstructing the surface of dental crowns. Hwang et al. [20] presented a crown reconstruction method based on a conditional generative adversarial network (CGAN). Yuan et al. [21] proposed a CGAN incorporating perceptual feature loss for dental occlusal surface reconstruction. Similarly, DAIS [22] model is the first to apply deep learning to the 3D design task of complex oral prosthesis, and successfully solves the problems of inheritance of experts high-quality design experience and intelligent design of prosthesis.

With a complex local anatomical morphology, such as dental fissures, cusps, and pits, the natural crown surface is the most important morphological feature of the occlusal surface and plays a crucial role in normal dental occlusion. To address the lack of detailed occlusal pit and fissure features in existing dental restoration methods and to improving the quality of crown restoration, this work proposes an image generation technology for functional occlusal pits and fissures based on the CGAN. First, a heuristic search method was adopted to extract dental pits and fissures, and an orthogonal projection method was employed to construct a tooth depth image dataset. Then, a functional occlusal pit and fissure extraction network was built based on a CGAN framework, and the images of missing dental pits and fissures were reconstructed by introducing jaw position spatial constraints, an *L*1 loss function, and a perceptual loss function to guide the optimization and improvement of the model parameters. Finally, the quality of the generated images was assessed using image quality evaluation metrics, and the reconstructed crown was compared with expert data to determine the effectiveness of our method.

## 2. Methods

### 2.1 Dental pit and fissure extraction based on a heuristic search method

The quality of neural network training results is associated with the quality of the datasets. In this study, a 3D dental pit and fissure model was projected onto a 2D plane to establish network training datasets. Whether the crown occlusal pit and fissure model can be extracted quickly and accurately determines the quantity and quality of the network training datasets. In this study, a 3D model of occlusal pits and fissures was extracted using a heuristic search method. The distances between vertices and the direction and curvature of the tooth mesh were used to define the heuristic function. The path with the minimum heuristic function cost between two vertices was calculated as the biometric segmentation line for extracting dental pits and fissures. The heuristic function was defined as follows:

$$f(n)=f_{dir1}+f_{dir2}+f_{D}+f_{C}$$ (1)

where *f*_*dir*1_ is the included angle between the current search direction and the previous search direction, with a smaller angle value indicating a smoother segmentation line; *f*_*dir*2_ refers to the included angle between the next search direction and the direction of the starting and ending points; *f*_*D*_ is the distance between the next search point and the starting search point (functions *f*_*dir*2_ and *f*_*D*_ can ensure that the search segmentation line is closed); and *f*_*C*_ represents the curvature difference between the front and rear search points, whose value can ensure that the search path describes the occlusal pit and fissure features to the maximum extent. Each cost function is assigned a weight factor, so Eq (1) can be rewritten as

$$f(n)=\lambda_{a}f_{dir1}+\lambda_{b}f_{dir2}+\lambda_{c}f_{D}+\lambda_{d}f_{C}$$ (2)

where *λ*_*a*_, *λ*_*b*_, *λ*_*c*_ and *λ*_*d*_ are the weight factors of the four cost functions, which were set to 0.75, 0.85, 0.85, and 0.55 based on actual tests and analysis.

**Fig 1:**
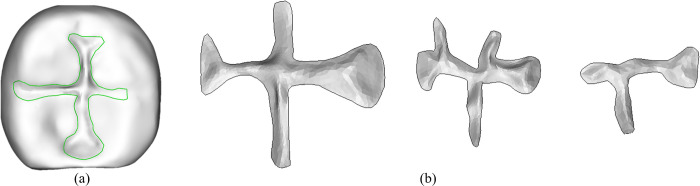
Extraction of occlusal pits and fissures. The dental pits and fissures feature lines obtained through heuristic search not only closely approximate the pit and fissure margins but also reduce a significant amount of manual interaction, thus improving the efficiency of dataset construction.

As shown in Fig 1(A), the feature lines of dental pits and fissures obtained from our heuristic search were close to the pit and fissure margins, and the overall morphology was relatively smooth. The extraction results (Fig 1(B)) illustrate that this method can quickly and accurately extract a 3D model of dental pits and fissures.

### 2.2 Construction of a tooth depth map using an orthogonal projection method combined with image entropy

**Fig 2:**
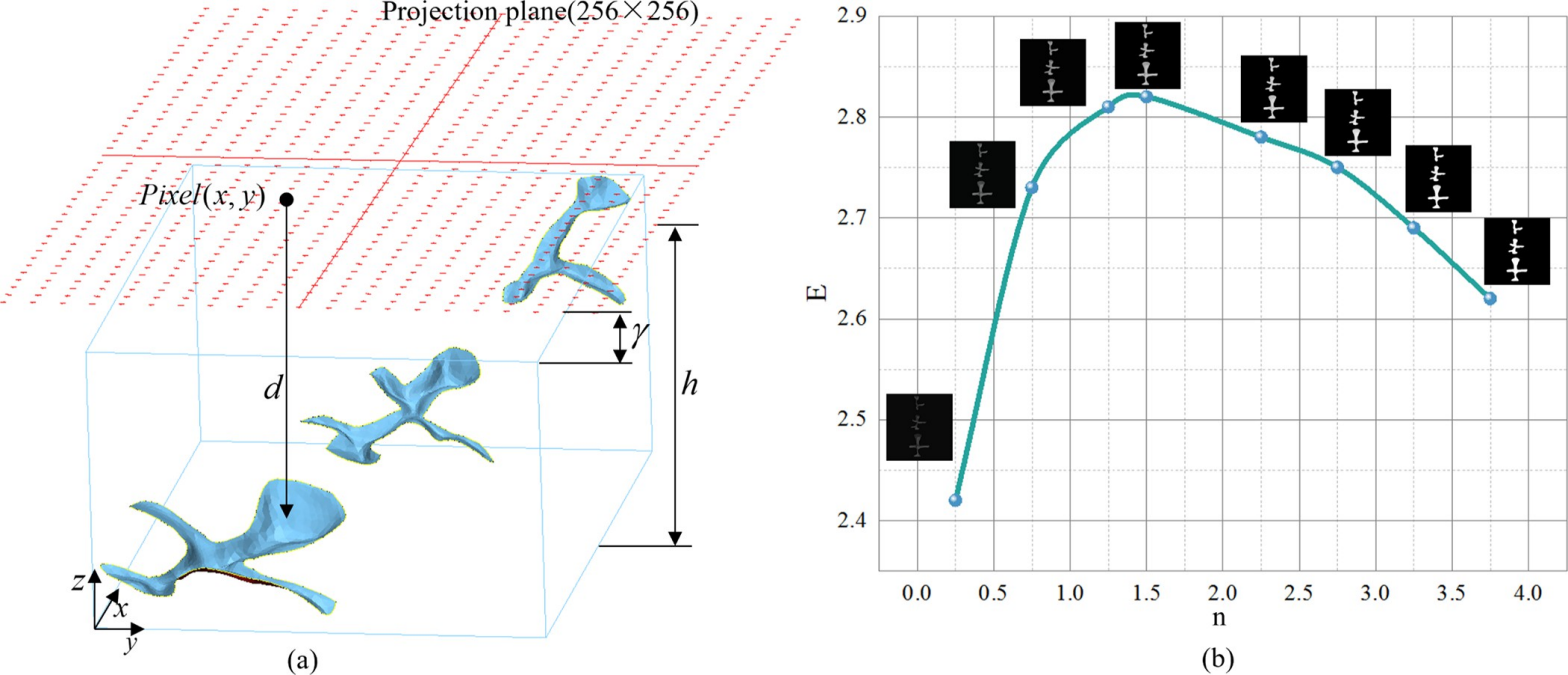
Tooth depth map construction and image entropy change curve. (a) Construction of the tooth depth map through orthogonal projection. (b) Image entropy change curve. Using a projection plane of size 256×256, the vertical distance (*d*) from the projection plane to the surface of the tooth model is computed, resulting in the pixel value of that point on the depth image.

GANs [23] are particularly good at processing 2D images, but the data in this study were obtained directly from a 3D tooth mesh model, so an orthogonal projection method was applied to project the 3D tooth model onto a 2D plane to convert it into a 2D depth image, which was used for network training. To improve the resolution of grayscale images of dental pits and fissures, the number of projection points was increased using a ray-intersection method. Before projection, the coordinate system was normalized by adjusting the posture of the tooth model via artificial interaction to ensure that the tooth model could be completely mapped to the 2D plane. As shown in Fig 2(A), the resolution of the tooth depth map constructed in this study was 256×256, with the pixel value indicating the vertical distance between the projection plane and the dental occlusal surface projection point; the smaller the distance between the projection point and the projection plane was, the higher the pixel value of that point on the depth map was.

The depth map was plotted through the following steps. First, a bounding box for the 3D tooth model was calculated, and the center of the bounding box perpendicular to the plane in the Z-axis direction was found, centered on which the projection grid was set at a distance *γ* above the 3D tooth model. The projection point was constructed using a ray-intersection method, and the vertical distance *d* from point *Pixel*(*x*,*y*) to the tooth model surface was calculated. Next, the limit plane at distance *h* from the projection plane was defined, and the pixel values of the points on the tooth model surface beyond this plane were set to 0 after projection. For the points on the model surface within the limit plane, their vertical distances from the projection surface were transformed into pixel values ranging from 0 to 255 according to the formula below:

$$Pixel(x,y)=255-\frac{255d^{n}}{h^{n}}$$ (3)

where *Pixel*(*x*,*y*) is the grayscale value of the pixel point to be projected; *d* specifies the vertical distance from the projection point to the tooth model; *h* refers to the distance from the projection plane to the limit plane, which was set to 6.5 in this study according to actual test results; and *n* is an image enhancement coefficient, which is correlated with the brightness and quality of the grayscale image. In this study, the richness of information carried by the grayscale images obtained through projection was evaluated using image entropy to define the value of *n*. The image entropy was calculated as

$$E=-\sum_{i=0}^{n}P(i)\log_{2}P(i)$$ (4)

where *E* is the image entropy; *P*(*i*) is the occurrence probability of pixel value *i* in the image; and *n* denotes the range of grayscale values (0–255).

The effect of the *n* value on the image entropy *E* is presented by the curve in Fig 2(B). A higher *E* value indicated a greater deviation from the peak grayscale area of the image histogram in the image and richer image information content. It can be seen from the curve that the image entropy *E* first increased and then decreased with greater *n* values, reaching its peak at *n* = 1.4. Therefore, *n* = 1.4 was selected to construct the tooth depth map dataset.

### 2.3 Construction of a functional occlusal pit and fissure extraction network model

**Fig 3:**
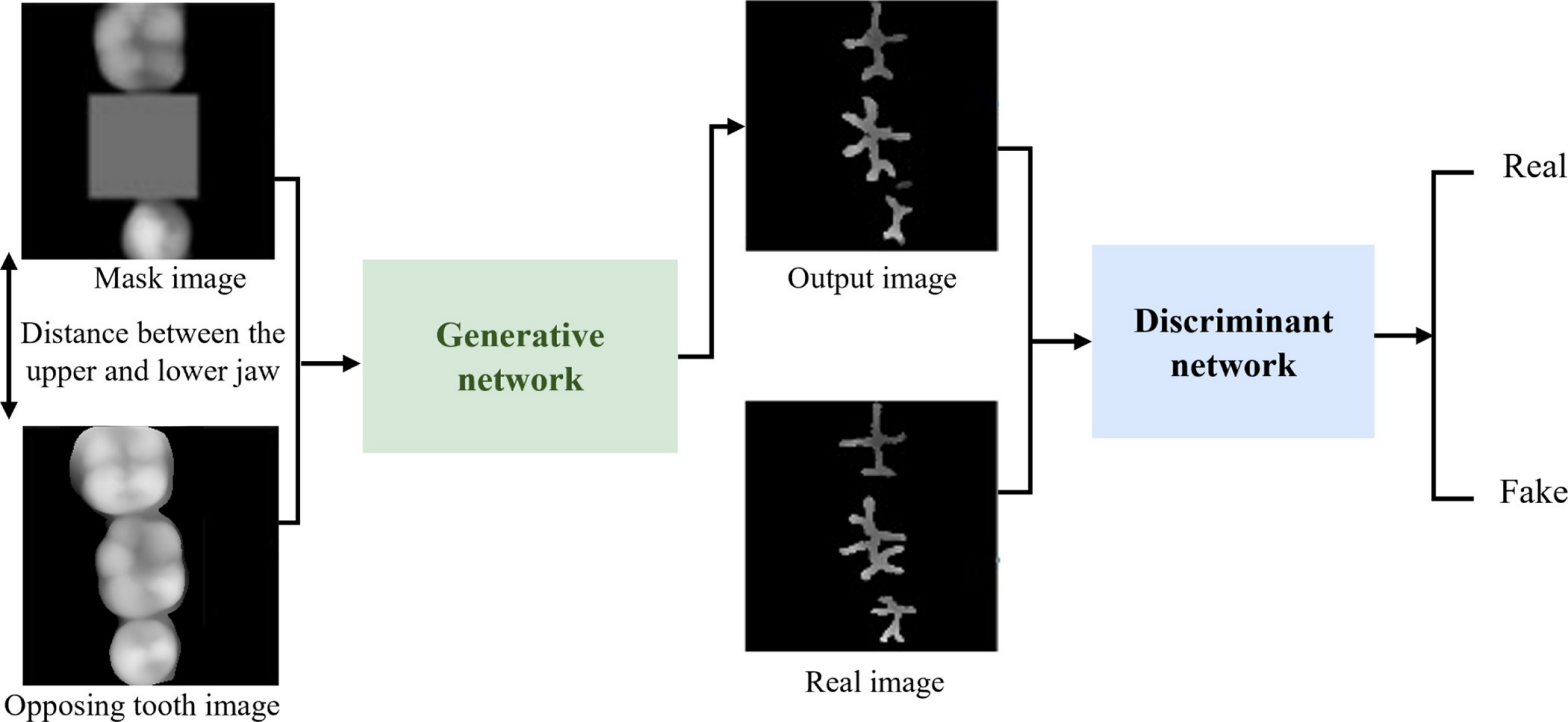
Network structure diagram. The network consists of generative model and discriminant model. During the training process, the generative model or discriminant model was fixed, and the other model was trained so the two models constantly confronted each other and updated their weights with alternating iterations until the two models reached a balance.

CGANs [24] have been widely applied because of their ability to input additional conditional information based on GANs, with stable generative models and controllable training processes. pix2pix [25] is a typical CGAN framework that has been extensively utilized in various image transformation tasks. In this study, with the jaw spatial position as a constraint, a functional occlusal pit and fissure extraction network model was prepared based on a pix2pix network (Fig 3). The network training process was supervised by defining a loss function to make the image generated by the generative model closer to the real data, while the discriminant model maximized the correct distinction between the real image and the generated image.

#### 2.3.1 Generative network and discriminant model

**Fig 4:**
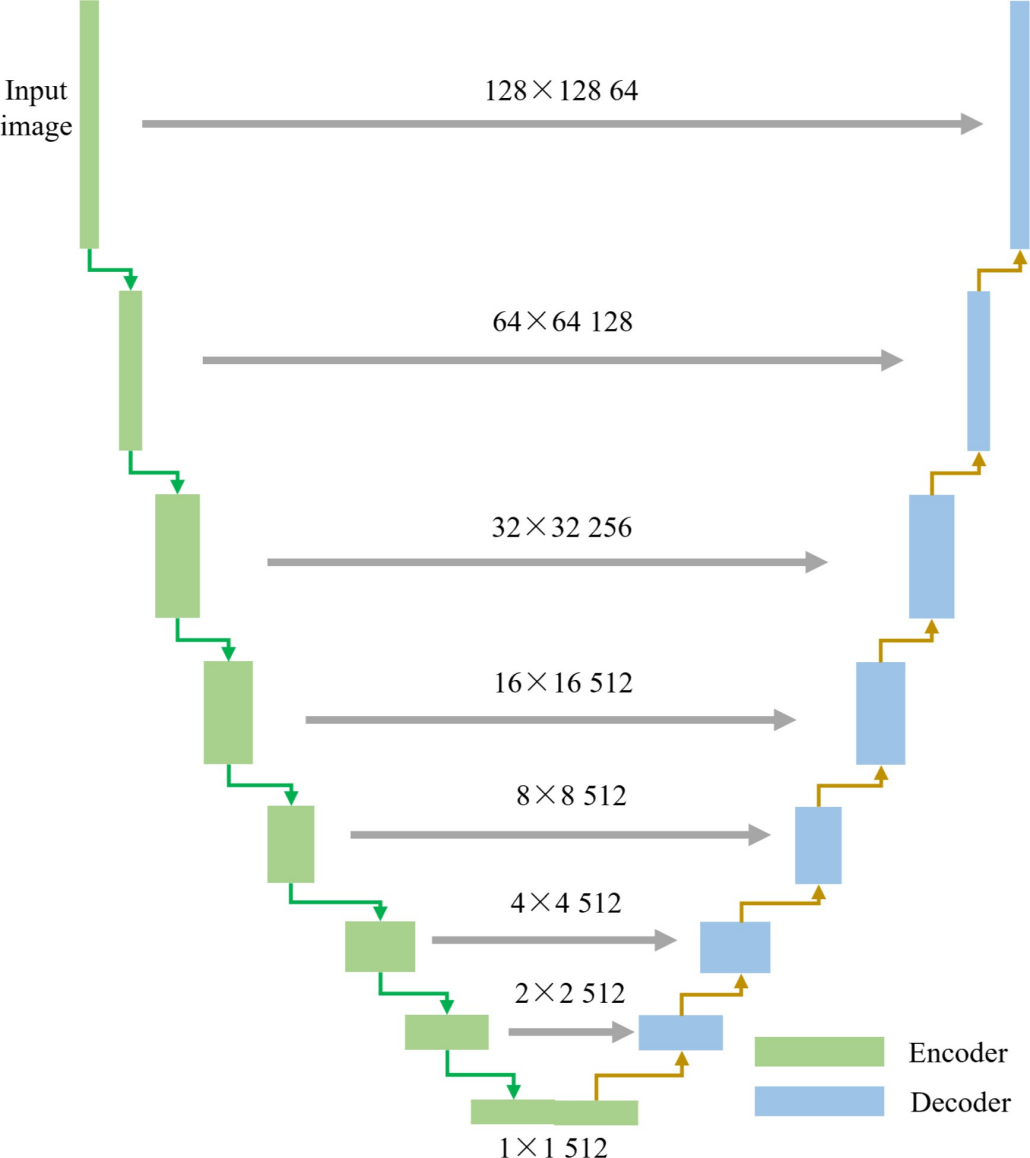
Generative network model structure. The encoder and decoder have a stride of 2, and the convolutional kernel size is 4×4.

As shown in Fig 4, the generative network model adopted the U-Net fully convolutional structure with symmetric distribution [26]. The network was composed of a coding structure [including convolution, batch normalization and activation function (LReLU)] and a decoding structure [including convolution, batch normalization, and activation function (ReLU)]. To maintain the detailed image features during training, the i^th^ hidden layer of the coding structure was connected to the (n-i)^th^ hidden layer of the decoding structure, and a skip connection fused the features of both the downsampling network and upsampling network, yielding accurate tooth image pixel information.

**Fig 5:**
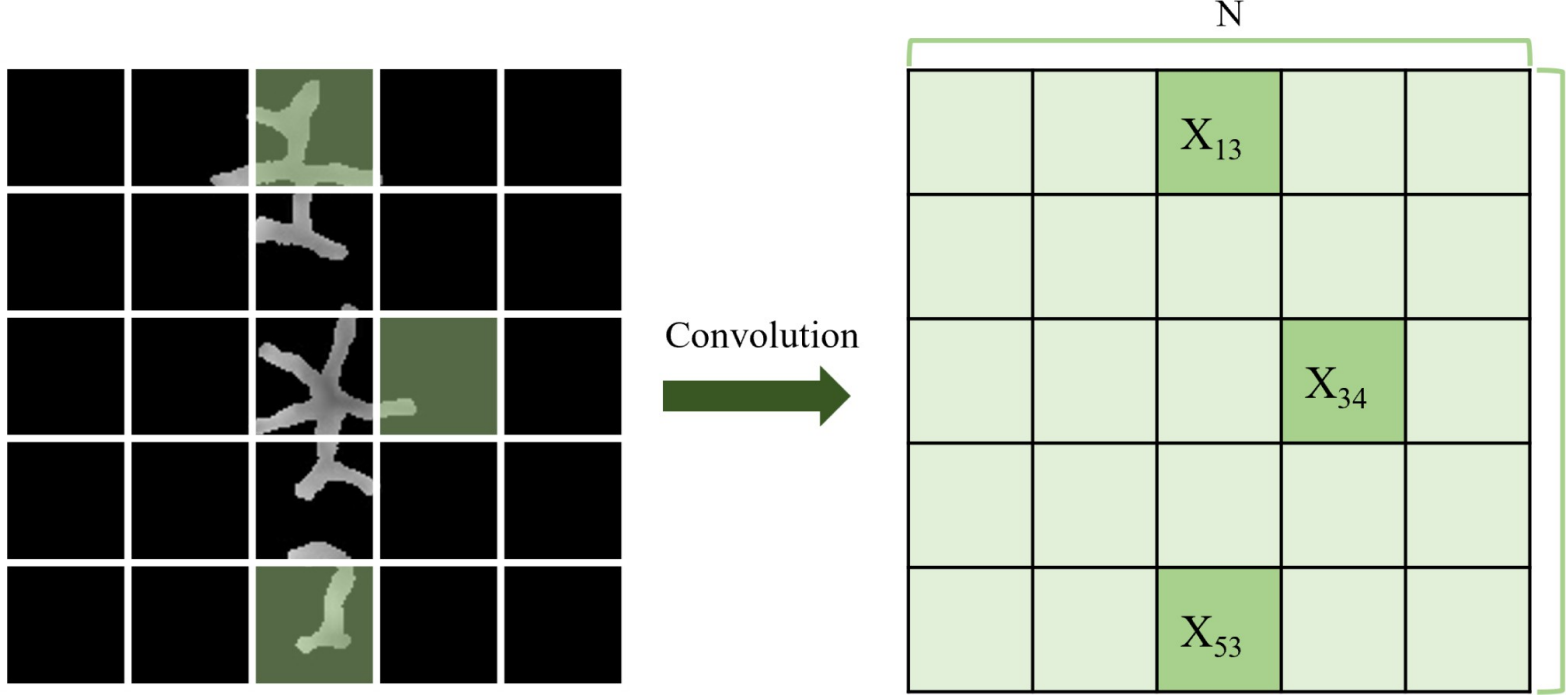
PatchGAN receptive field mechanism. The discriminative network divides the input data into N×N patch blocks and performs convolution on each patch block, resulting in a response matrix X, which serves as the output of the discriminative network. This mechanism combines both the overall features and local features of the occlusal surface depth image, making the judgment of the generated network model’s output more reliable.

The discriminant network in the GAN needs to determine the authenticity of the input pit and fissure images. The output of the previous discriminant network is a probability value from the target data distribution, which serves as a scalar ranging from 0 to 1 but cannot reflect the local image features. A dental occlusal surface has numerous local detailed features and complicated images, so this method has limitations. In this study, the PatchGAN [27] framework was applied to construct a discriminant network model that output an N×N matrix, each element of which represented the discrimination result of the corresponding image area (Fig 5). The discriminant network divided the input images into N×N receptive fields, called patch blocks. Then, each patch block was subjected to a convolution operation to obtain the corresponding matrix X, where each element X_ij_ was the authenticity discrimination probability value on the corresponding area. Finally, all the probability values were averaged as the discriminant network output. This method considered the impact of different parts of the images, fused the local and overall image features, extracted local image features, and enhanced the reliability of the generative network model, thereby producing pit and fissure images with high resolution and quality.

**Fig 6:**
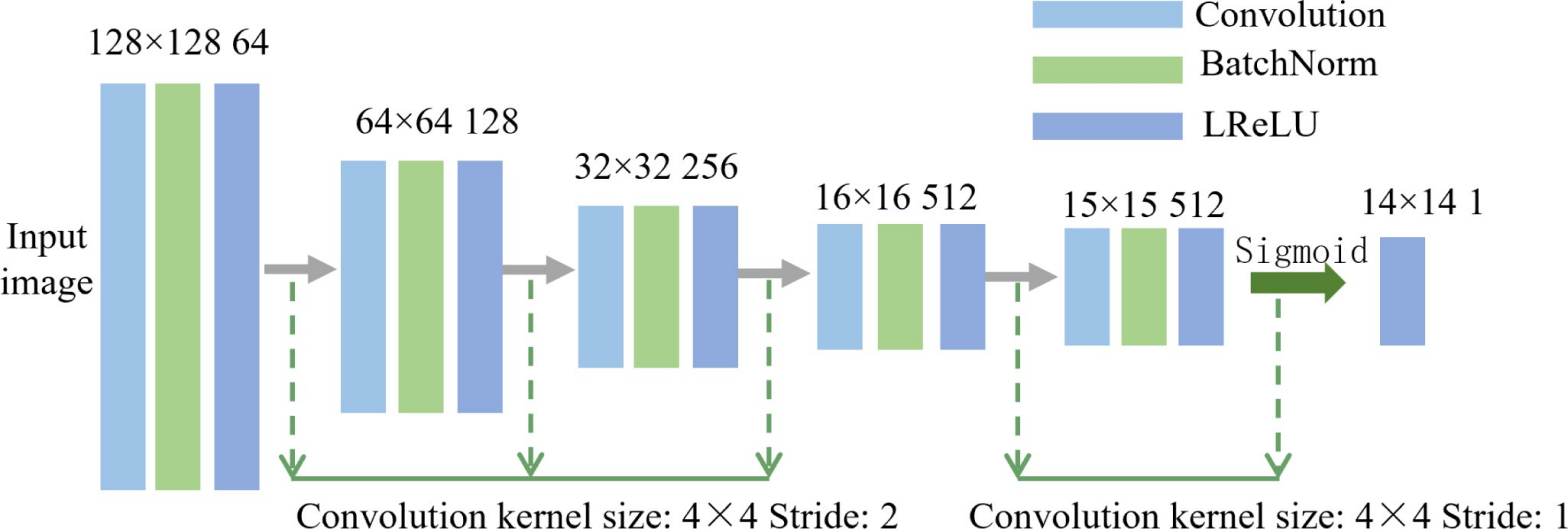
Discriminant network model structure.

As shown in Fig 6, the discriminant network had a convolutional structure, with convolution + batch normalization + activation function (LReLU) and the convolution kernel size was 4 × 4. In the last network layer, the sigmoid function was used as the patch matrix classification function.

#### 2.3.2 Network model objective function

During training, the generative network and the discriminant network confronted each other and continuously optimized their corresponding loss functions. The key to dental restoration lies in the construction of a normal occlusal relationship between the occlusal surfaces of the upper and lower teeth. As a result, we used the data of opposing teeth as a jaw space spatial constraint to guide the generative network model to produce the morphology of occlusal pits and fissures with a correct occlusal relationship and good contact distribution. The objective function was

$$L_{CGAN}(G,D)=E_{x,op,y}[\log D(x,op,y)]+E_{x,op,z}[\log(1-D(G(x,op,z)))]$$ (5)

where *x* is the mask image of the missing tooth; *op* indicates the image of its opposing tooth; *y* refers to the image of the target occlusal pits and fissures; and *z* is Gaussian noise.

The *L*1 loss function [28] is fairly common in deep learning. It is also known as the least absolute error. Specifically, the *L*1 loss function is rarely affected by a large error, remaining stable in such situations. In this study, the *L*1 loss function was introduced as a neural network constraint to guide the network model training, which was beneficial to the reconstruction of occlusal pit and fissure images. It is defined as follows:

$$L1(G)=E_{x,y,z}[\|y-G(x,op,z)\|]$$ (6)

where *x* denotes the image of the input missing tooth; *op* is the image of the tooth opposing the missing tooth; *z* is Gaussian noise; and *y* specifies the target image. The *L*1 loss function is defined as the least absolute error between the generated image and the real image.

Occlusal pits and fissures have many complex, personalized morphologies [29]. To further enhance the reconstruction capability of the network model for pit and fissure images, we employed the hidden layer in the discriminant network model to measure the deviation of perceptual features [30] between the generated and target images of occlusal pits and fissures so that the discriminant network model could better evaluate the probability that the data generated by the generative network model came from the real data. The introduced perceptual loss function was

$$S_{i}(G,y)=\frac{1}{C_{i}H_{i}W_{i}}\|h_{i}(y)-h_{i}(G)\|$$ (7)

$$L_{P}(G)=E_{x,y,z}[\sum_{i}\lambda_{i}S_{i}(G,y)]$$ (8)

$$L_{P}(D)=E_{x,y,z}[\max\{0,m-\sum_{i}\lambda_{i}S_{i}(G,y)\}]$$ (9)

where *S*_*i*_(*G*,*y*) is the deviation between the images of occlusal pits and fissures generated by the network and the target images in the i^th^ hidden layer of the discriminant network; *C*_*i*_, *H*_*i*_, and *W*_*i*_ refer to the channel number, height and width of the i^th^ hidden layer, respectively; *L*_*P*_(*G*) and *L*_*P*_(*D*) denote the constraint equations of the perceptual loss in the generative network and discriminant network, respectively, and *m* signifies the boundary value of high-dimensional feature loss in the discriminant network.

After combining the *L*1 loss function and perceptual feature loss function, the objective function of the functional occlusal pit and fissure extraction network becomes

$$L(G)=\arg\;\underset{G}{\min}L_{CGAN}(G,D)+\lambda_{1}L1(G)+\lambda_{PG}L_{P}(G)$$ (10)

$$L(D)=\arg\;\underset{D}{\max}L_{CGAN}(G,D)+\lambda_{PD}L_{P}(D)$$ (11)

where *L*(*G*) and *L*(*D*) refer to the perceptual feature losses of the generative network and the discriminant network, *λ*_*PG*_ and *λ*_*PD*_ represent the weight parameters in the generative network and the discriminant network of perceptual loss, respectively.

## 3. Results

### 3.1 Experimental datasets and parameter settings

**Fig 7:**
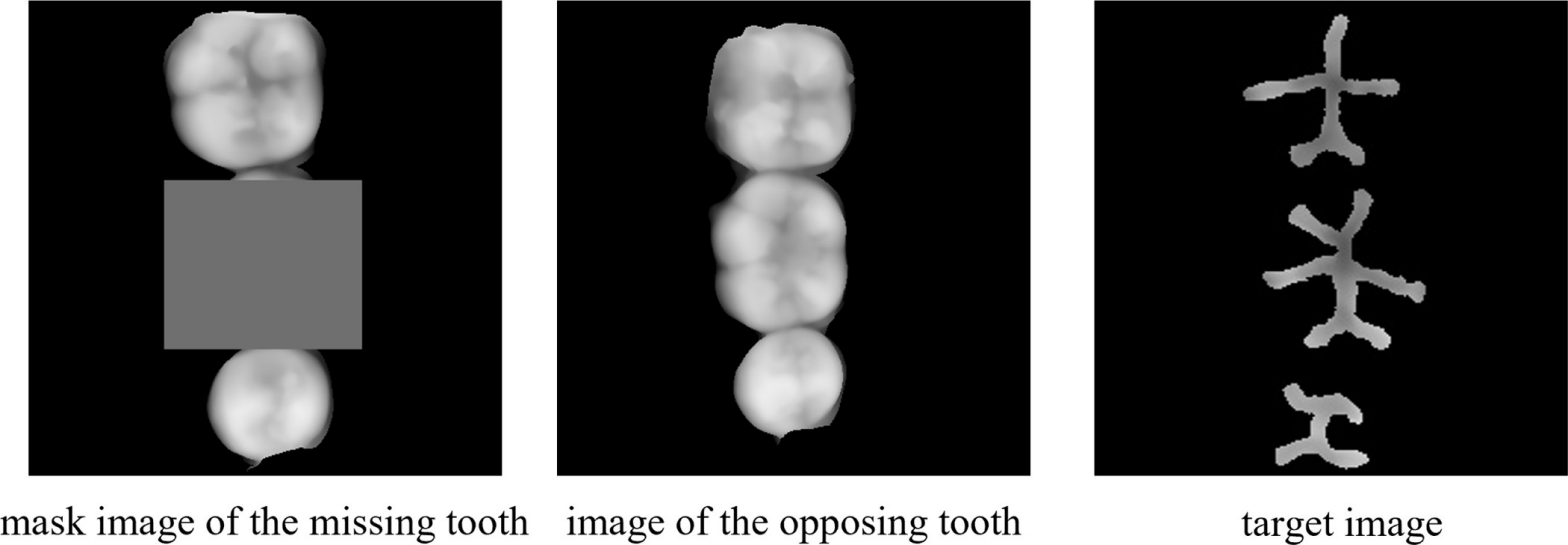
Network training data set.

A computer configured with an Intel Xeon Platinum 8168 CPU and a GeForce GTX 1080Ti GPU (12 GB video memory) was used as the hardware for the experiments, and the Windows 10 operating system, PyTorch 1.2.0, CUDA 8.0, and Python 3.5 made up the network training environment. Given the basically similar morphology of homonym teeth, the premolars and molars, which have a high defect rate in the clinic, were taken as the subjects in this study. A dataset containing 400 pairs of tooth images was built using the method introduced in Section 2.2, of which 300 pairs of images served as the training set and 100 pairs of images functioned as the test set. Each pair of images included a mask image of the missing tooth, an image of the opposing tooth, and a target image of extracted occlusal pits and fissures (Fig 7).

The important parameters of the network are listed in Table 1.

**Table 1:** Functional occlusal pit and fissure extraction network parameter settings.

Parameter	Value	Parameter	Value
Initial learning rate	0.0002	*L*1 loss function weight, *λ*_1_	100
Momentum parameter, *β*_1_	0.5	Generative network perceptual loss weight, *λ*_*PG*_	50
Momentum parameter, *β*_2_	0.999	Discriminant network perceptual loss weight, *λ*_*PD*_	20
Batch size	1	Perceptual loss weight of layer 2, *λ*_*la*2_	1
Feature loss boundary value, *m*	0.35	Perceptual loss weight of layer 3, *λ*_*la*3_	2
Training epochs	200	Perceptual loss weight of layer 4, *λ*_*la*4_	2

### 3.2 Network training results

Considering the difference in loss functions during network model construction, three groups of experiments were conducted in this study. In group 1, only the jaw position spatial constraint was applied to guide the generation of occlusal pits and fissures, and the *L*1 loss function was introduced in group 2 based on group 1. In group 3, the perceptual feature loss function was employed based on group 2 to improve the reconstruction capability of the network for the detailed features of the occlusal pits and fissures (Table 2).

**Table 2:** Settings of different experiment groups.

Loss function Group	Spatial constraint of jaw position	*L*1 loss function	Perceptual feature loss function
Group 1	✓		
Group 2	✓	✓	
Group 3	✓	✓	✓

**Fig 8:**
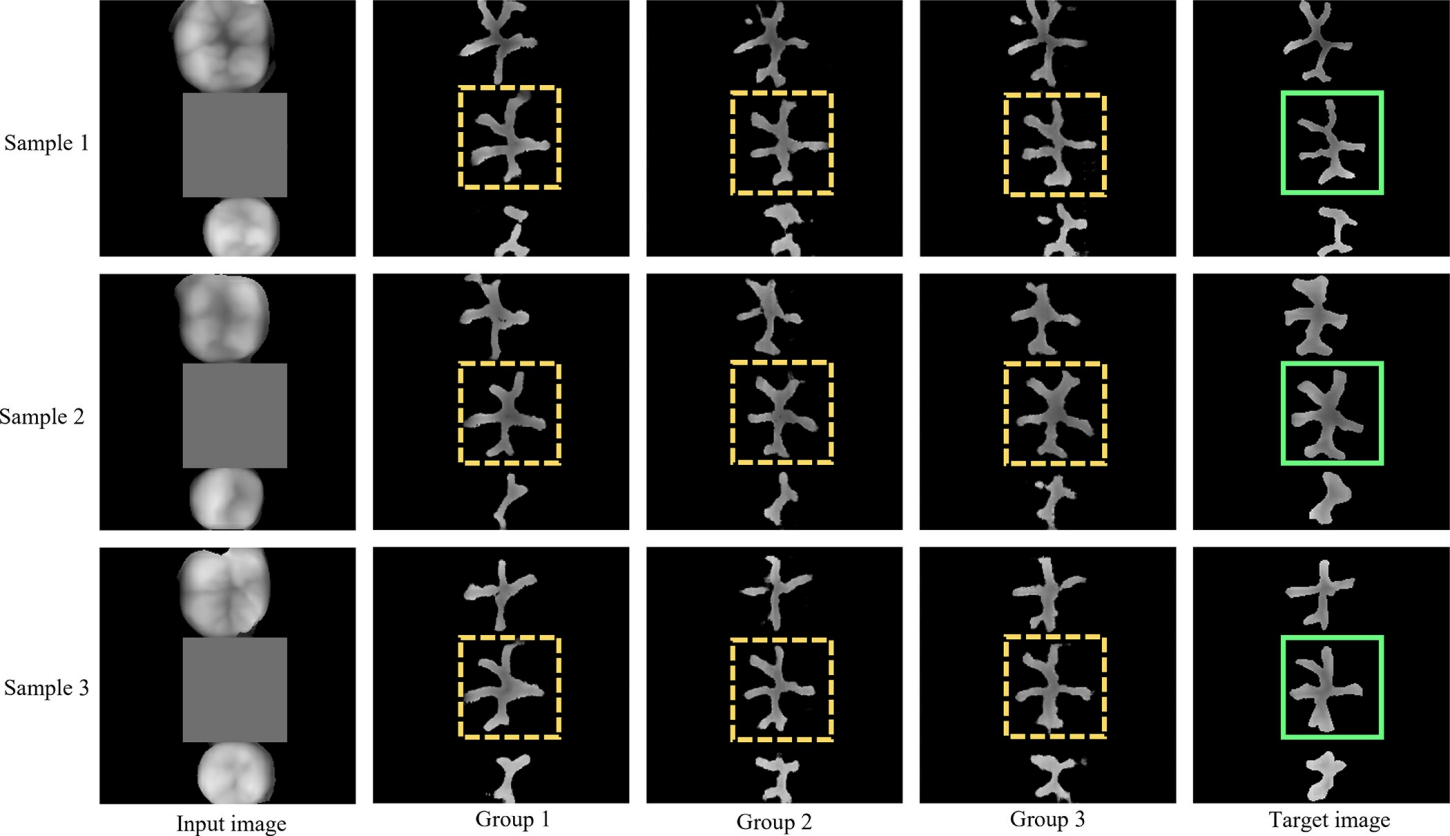
Network training results for different experimental groups.

After network training, the three groups of network results were tested separately. In Fig 8, the first column shows the input images for network testing; the second, third, and fourth columns present the network generation results in groups 1, 2, and 3, respectively; and the last column displays the target images. The network-generated images of the dental pits and fissures to be restored are marked with dotted boxes in each figure. The results showed that the pit and fissure images were successfully generated via the three groups of experiments. Specifically, the images generated in group 1 differed greatly from the target image. After adding the *L*1 loss function, the images generated in group 2 improved upon the earlier images, and the morphology of pits and fissures was relatively close to their target morphology. When the perceptual loss function was added, the morphology of pits and fissures best approximated the target morphology of pits and fissures, suggesting that the *L*1 and perceptual loss functions benefitted the network for a better reconstruction of the morphology of occlusal pits and fissures and optimal performance.

## 4. Discussion

### 4.1 Image quality assessment

In this study, the quality of occlusal pit and fissure images generated by the network was evaluated using two image quality evaluation metrics, the peak signal-to-noise ratio (PSNR) [31] and structural similarity index measure(SSIM) [32], which are defined in Eq (13) and Eq (14), respectively:

$$MSE=\frac{1}{mn}\sum_{i=0}^{m-1}\sum_{j=0}^{n-1}{\left[X(i,j)-Y(i,j)\right]}^{2}$$ (12)

$$PSNR=10*\log_{10}(\frac{MAX^{2}}{MSE})$$ (13)

where *MSE* is the mean square error; *m* and *n* are the width and height of the image, respectively; *X*(*i*,*j*) and *Y*(*i*,*j*) are the pixel values in the target image (expert data) and generated image, respectively; and *MAX* is the maximum pixel value of the image, which was set to 255.

$$SSIM(x,y)=\frac{\left(2\mu_{x}\mu_{y}+c_{1})(2\sigma_{xy}+c_{2}\right)}{\left(\mu_{x}{}^{2}+\mu_{y}{}^{2}+c_{1})(\sigma_{x}{}^{2}+\sigma_{y}{}^{2}+c_{2}\right)}$$ (14)

where *μ*_*x*_ and *μ*_*y*_ represent the means of images *x* and *y*, respectively; *σ*_*xy*_ denotes the covariance of images *x* and y; *σ*_*x*_^2^ and *σ*_*y*_^2^ are the variances of images *x* and *y*, respectively; and *C*_1_ and *C*_2_ are constants set to 6.5 and 58.5, respectively.

The PSNR and SSIM values of the three samples in Fig 8 from the three groups of experiments are listed in Table 3. The higher the two values were, the closer the occlusal pit and fissure image generated by the network was to the target image. It can be seen from the table that the PSNR and SSIM values in group 3 were better than those in groups 1 and 2, implying that the ability of the network to synthesize the occlusal pit and fissure image was enhanced after the introduction of the *L*1 loss and perceptual losses.

**Table 3:** Quality evaluation values of occlusal pit and fissure images.

Sample	Sample 1			Sample 2			Sample 3		
Group	Group 1	Group 2	Group 3	Group 1	Group 2	Group 3	Group 1	Group 2	Group 3
PSNR	20.91	19.43	**20.35**	21.34	21.85	**22.45**	22.79	22.25	**23.38**
SSIM	0.7454	0.7951	**0.8263**	0.7427	0.7865	**0.8412**	0.7241	0.7943	**0.8296**

### 4.2 Comparison to other methods

**Fig 9:**
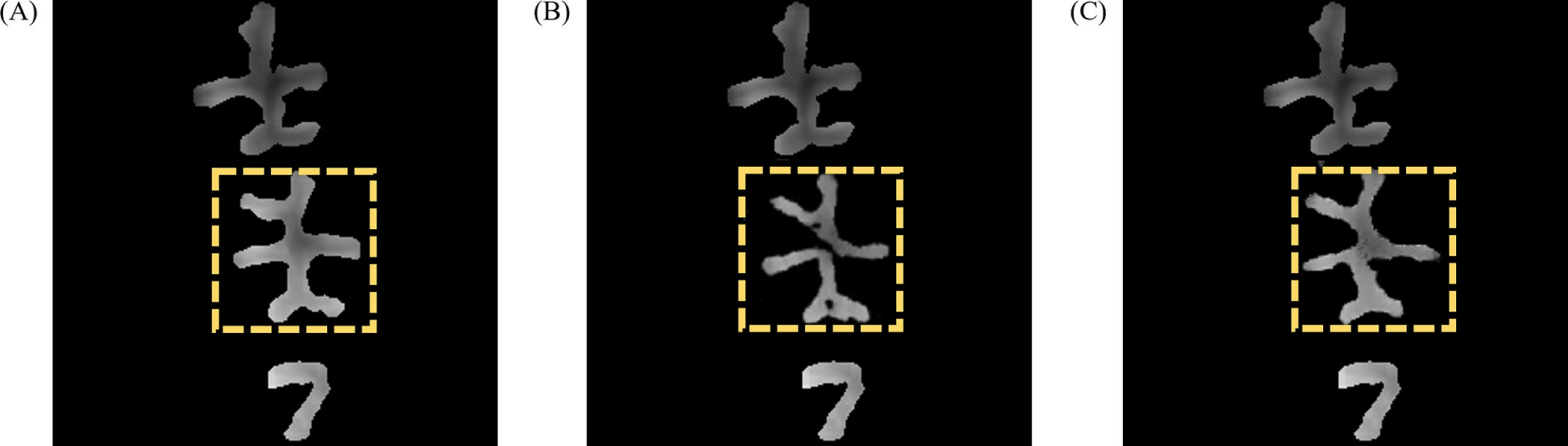
Comparison of different methods. (A) Target image. (B) Tian’s method. (C) Ours.

To further demonstrate the advancement of the method proposed in this article, we compare the pit and fissure images generated by our network with Tian’s method [33] in this section. We selected tooth samples from the same patient to generate images. Fig 9 shows the generated results, where Fig 9(A) is the target image and Fig 9(B) is the image generated using Tian’s method, which only considers the jaw position spatial constraint, resulting in a relatively large difference between the generated image and the target image. Fig 9(C) is the image generated using our method, which is more similar to the target image. This is because we introduced a perceptual feature loss function to measure the perceptual feature deviation between the generated occlusal pit and fissure image and the target image, allowing the discriminative network model to better determine the probability of the generated network model data coming from real data, further enhancing the network model’s ability to reconstruct pit and fissure images.

### 4.3 Analysis of crown restoration quality

Although a crown designed based on a CAD system can restore the anatomical morphology of the occlusal surface, the surface changes among the detailed features of pits and fissures are relatively flat, and there is a lack of significant pit and fissure morphology. To ensure that the restored crown had a more personalized morphology while meeting the requirements for functional occlusion, the pixel values of the pit and fissure images were converted into 3D point cloud data through an inverse projection transformation based on the experiment in group 3. In addition, the point cloud was transformed into a mesh using a region growing method [34]. Finally, with the surface of the occlusal pits and fissures as the constraint, the central fissure region of the restored crown was deformed via a minimum-energy surface deformation method, thus enriching the surface features of the generated crown.

**Fig 10:**
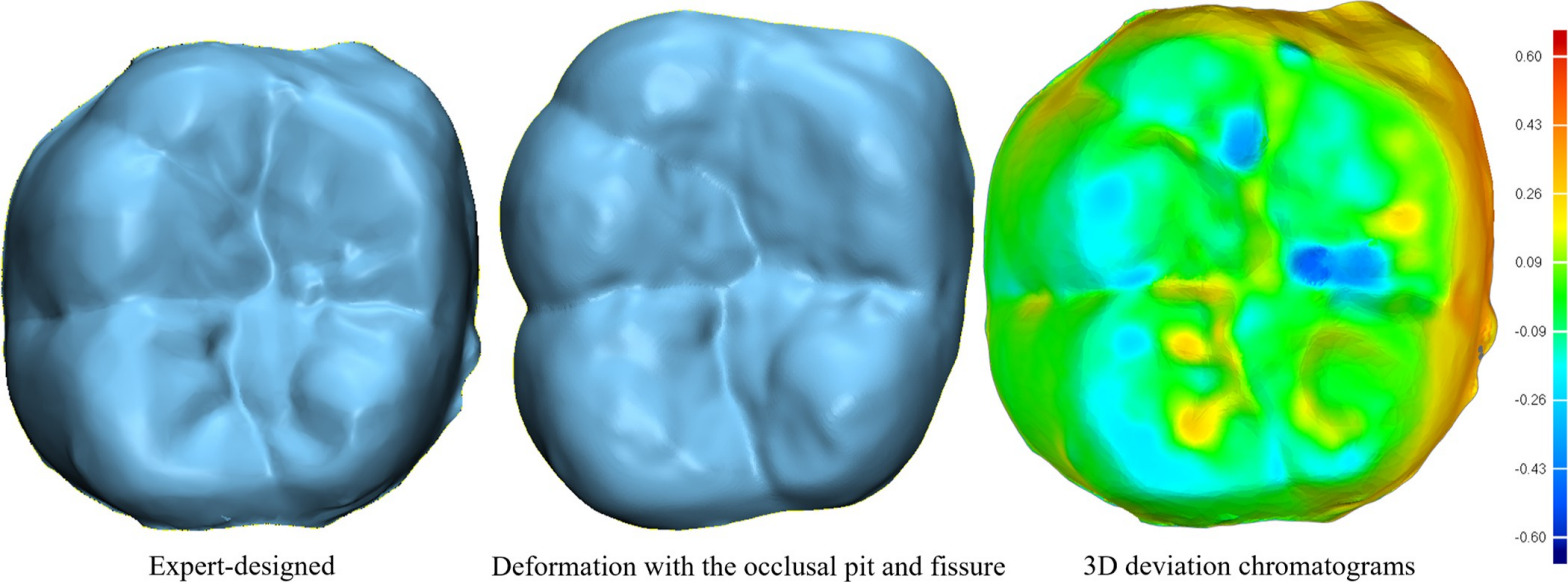
Occlusal surface reconstruction results and deviation analysis.

An expert-designed crown model and the crown model obtained from the local deformation with the occlusal pit and fissure model of the corresponding crown as the constraint, and the 3D deviation chromatograms between the models are displayed in Fig 10. According to the chromatograms, the two models highly resembled each other, with only a few areas having small deviations. Therefore, our model can meet the needs of clinical applications.

The deviation between the deformed model and the expert-designed model in Fig 10 was evaluated quantitatively. Both the standard deviation (0.1802 mm) and the root mean square error estimate(0.2140 mm) had low values, showing that using the network-generated occlusal pits and fissures as constraints provides a good crown occlusal surface deformation guide. As a result, the crowns after deformation were closer to the expert-designed crowns.

## 5. Conclusion

In this paper, we construct an occlusal surface pit and fissure generation network model based on CGAN. We utilize opposing tooth as the constraints and combined *L*1 loss and perceptual loss functions to enhance the network’s ability of reconstructing fissure images. This method is an improvement over existing approaches, addressing the issue of lacking local detailed crown surface features in current dental restoration methods. After applying various image quality evaluation methods, the results demonstrate that the generated images by this network effectively restore missing details of crown fissures. However, this method also has certain limitations in that it cannot directly generate three-dimensional models of dental crown occlusal surfaces. In the future, we will further expand the sample size of teeth, improve the generalization ability of the neural network, and explore three-dimensional data network design methods targeting different tooth positions and multiple diseased teeth.

## Supporting information

S1 Dataset (RAR)
